# Genomic aberrations relate early and advanced stage ovarian cancer

**DOI:** 10.1007/s13402-012-0077-5

**Published:** 2012-05-12

**Authors:** Afra Zaal, Wouter J. Peyrot, P. M. J. J. Berns, Maria E. L. van der Burg, Jan H. W. Veerbeek, J. Baptist Trimbos, Isabelle Cadron, Paul J. van Diest, Wessel N. van Wieringen, Oscar Krijgsman, Gerrit A. Meijer, Jurgen M. J. Piek, Petra J. Timmers, Ignace Vergote, René H. M. Verheijen, Bauke Ylstra, Ronald P. Zweemer, On behalf of the EORTC GCG Translational Research Group

**Affiliations:** 1 Department of Gynaecological Oncology, University Medical Center Utrecht, PO Box 85500, 3508 GA Utrecht, The Netherlands; 2 EORTC Gynaecological Cancer Group Translational Research Group chair, Avenue Mounierlaan 83/11, 1200 Brussels, Belgium; 3 Department of Medical Oncology, Erasmus MC; University Medical Center Rotterdam, PO Box 2040, 3000 CA Rotterdam, The Netherlands; 4 Study Coordinator EORTC-ACTION trial, Department of Gynaecological Oncology, Leiden University Medical Center, PO Box 9600, 2300 RC Leiden, The Netherlands; 5 Department of Gynecologic Oncology, University Hospitals Leuven, Herestraat 49, 3000 Leuven, Belgium; 6 Department of Pathology, University Medical Center Utrecht, PO Box 85500, 3508 GA Utrecht, The Netherlands; 7 Department of Biostatistics and Epidemiology, VU University Medical Center, PO Box 7057, 1007 MB Amsterdam, The Netherlands; 8 Department of Mathematics, VU University Medical Center, PO Box 7057, 1007 MB Amsterdam, The Netherlands; 9 Department of Pathology, VU University Medical Center, PO Box 7057, 1007 MB Amsterdam, The Netherlands; 10 Comprehensive Cancer Center South location TweeSteden hospital, Dr. Deelenlaan 5, 5042AD Tilburg, The Netherlands; 11 Department of Gynecology and Obstetrics, Maasstad Hospital, PO Box 9100 3007, AC Rotterdam, The Netherlands

**Keywords:** Oligonucleotide array, Ovarian neoplasms, Chromosome aberrations, Neoplasm staging, Prognosis

## Abstract

**Background:**

Because of the distinct clinical presentation of early and advanced stage ovarian cancer, we aim to clarify whether these disease entities are solely separated by time of diagnosis or whether they arise from distinct molecular events.

**Methods:**

Sixteen early and sixteen advanced stage ovarian carcinomas, matched for histological subtype and differentiation grade, were included. Genomic aberrations were compared for each early and advanced stage ovarian cancer by array comparative genomic hybridization. To study how the aberrations correlate to the clinical characteristics of the tumors we clustered tumors based on the genomic aberrations.

**Results:**

The genomic aberration patterns in advanced stage cancer equalled those in early stage, but were more frequent in advanced stage (*p* = 0.012). Unsupervised clustering based on genomic aberrations yielded two clusters that significantly discriminated early from advanced stage (*p* = 0.001), and that did differ significantly in survival (*p* = 0.002). These clusters however did give a more accurate prognosis than histological subtype or differentiation grade.

**Conclusion:**

This study indicates that advanced stage ovarian cancer either progresses from early stage or from a common precursor lesion but that they do not arise from distinct carcinogenic molecular events. Furthermore, we show that array comparative genomic hybridization has the potential to identify clinically distinct patients.

**Electronic supplementary material:**

The online version of this article (doi:10.1007/s13402-012-0077-5) contains supplementary material, which is available to authorized users.

## Introduction

Despite improved survival over the last decades, ovarian cancer is still the most lethal gynaecological malignancy in the Western world, with a 5-year overall survival of only 25 % for advanced stage [1, 2]. In contrast, patients diagnosed at an early stage have a good 5-year overall survival rate of 93 %. Early stage ovarian cancer, however, is only diagnosed in 19 % of the patients.

Like other malignancies, epithelial ovarian cancer presumably results from an accumulation of genomic aberrations [3], but the exact molecular pathways by which these tumors develop have not been fully elucidated [4, 5]. For other tumors, such as cervical and anal cancer, intraepithelial neoplasia is known to be a precursor lesion. However, no clear precursor lesion is known for ovarian cancer. Therefore, the first clinical entity to study carcinogenesis of ovarian cancer in patients is minimal localized cancer (FIGO stage I disease). As a small percentage of the patients is diagnosed with early stage ovarian cancer, it has been hypothesized that early and advanced stage ovarian cancers are two distinct subtypes. Early stage ovarian cancer (with a good prognosis) may represent a distinct biological entity with low metastatic potential and would thus arise through a different carcinogenic pathway. It has been suggested that these early cases are molecularly distinguishable from the advanced stage ovarian cancer with a worse prognosis [6].

Copy number change is one of the key features of genetic instability in human cancer and can be measured in formalin-fixed, paraffin embedded tissue (FFPE)[7]. To test the hypothesis that early and advanced stage ovarian cancers are distinct molecular pathologic entities, we used array comparative genomic hybridization (array CGH) to detect and compare genomic aberrations in matched early and advanced stage ovarian tumors. Furthermore, we study how the aberrations correlate to the clinical characteristics of the tumors[8].

## Materials and methods

### Patients and tumor tissue

FFPE primary ovarian tumor tissue of 52 early stage ovarian cancer patients (FIGO stage I), was available from 448 participants of the EORTC ‘Adjuvant ChemoTherapy in Ovarian Neoplasm’ (ACTION) trial [9–11]. Of these 52 samples, 17 early stage samples with high quality DNA were matched for histological subtype and grade with FIGO stage III–IV (advanced stage) ovarian cancer samples from the Departments of Pathology of the University Medical Center Utrecht and VU University Medical Center Amsterdam, The Netherlands. FIGO stage was determined by optimal staging in all but four of the patients with advanced stage cancer. Staging was optimal (*n* = 7), modified (*n* = 3) or minimal (*n* = 7) as previously described [9]. In short, optimal staging consisted of inspection and palpation of all peritoneal surfaces; biopsies of any suspect lesions for metastases; peritoneal washing; infracolic omentectomy; (blind) biopsies of right hemidiaphragm, of right and left paracolic gutter, of pelvic sidewalls, of ovarian fossa, of bladder peritoneum, and of cul-de-sac; sampling of iliac and periaortic lymph nodes. Modified comprised of everything between optimal and minimal staging. Minimal staging was only inspection and palpation of all peritoneal surfaces and the retroperitoneal area; biopsies of any suspect lesions for metastases; peritoneal washing and infracolic omentectomy.

All samples were revised by an experienced gynaecological/oncological pathologist (PvD). The tumor percentage was defined by the pathologist per individual case; mean 77.8 % (SD 7.5), median 80 % range [65–95]. Samples were processed anonymously in accordance with institutional ethical guidelines, and follow up was retrieved through the EORTC ACTION trial database and the hospital information system for the early and advanced stage tumors respectively. Overall and progression free survival times were calculated from the time of randomization (within 6 weeks following staging) or the date of surgery for the early and advanced stage tumors respectively.

The age range was 27–80 years with a mean of 52 years for early and 62 years for advanced stage carcinomas. In six cases, an exact match for grade was unavailable. In these cases we matched with one grade difference being a grade 1 early with grade 2 advanced (*n* = 4), and a grade 2 early with grade 3 advanced (*n* = 2) (Table 1).

**Table 1:** Characteristics of all 32 patients included in this study. Overall (OS) and progression free survival (PFS) are calculated from time of randomization in the early (T102-T118) and time of diagnosis in advanced stage group (T302-T318). Histological subtypes are indicated as clearcell (C), endometrioid (E), mucinous (M) or serous (S). Adjuvant Chemotherapy (CT) was administered to a subset of patients. The results of the unsupervised clustering analysis are displayed as cluster A or B

Sample	Age	Stage	Type	Grade	CT	Staging	Status	OS (month)	Progression	PFS (months)	Cluster
T102	70	Ic capsule ruptured	C	2	no	optimal	Alive	42	no	42	A
T103	61	Ic capsule ruptured	C	3	yes	optimal	Alive	154	no	154	A
T104	61	Ia	C	3	yes	optimal	Dead	13	yes	10	A
T105	47	Ia	C	3	no	minimal	Alive	136	no	136	A
T106	64	Ic capsule ruptured	C	3	no	optimal	Alive	39	no	39	A
T108	41	Ic capsule ruptured	E	1	yes	minimal	Alive	73	no	73	A
T109	55	Ib	E	1	yes	minimal	Alive	137	no	137	A
T110	64	Ic ascites positive	E	1	no	modified	Alive	49	yes	49	A
T111	46	Ic ovarian surface	E	2	no	minimal	Alive	146	no	146	A
T112	53	Ic capsule ruptured	E	2	yes	minimal	Alive	44	no	44	A
T113	42	Ic capsule ruptured	M	1	no	optimal	Alive	69	no	69	A
T114	35	Ia	M	2	no	optimal	Alive	152	no	152	A
T115	27	Ia	S	1	yes	minimal	Alive	65	no	65	A
T116	62	Ia	S	1	no	minimal	Alive	59	no	59	B
T117	50	Ic ascites positive	S	1	no	modified	Dead	61	yes	7	B
T118	53	Ia ovarian surface	S	3	no	optimal	Alive	175	no	175	A
T302	47	III	C	2	yes	optimal	Dead	17	yes	17	B
T303	57	IV	C	3	yes	incomplete	Dead	9	yes	8	B
T304	51	III	C	3	yes	incomplete	Dead	14	yes	14	A
T305	74	IIIb	C	3	yes	optimal	Dead	60	yes	47	B
T306	48	IIIa	C	3	yes	optimal	Alive	104	no	104	B
T308	45	IV	E	2	yes	optimal	Dead	19	yes	9	B
T309	47	IIIc	E	2	yes	optimal	Dead	41	yes	21	B
T310	74	IIIb	E	2	yes	optimal	Dead	57	yes	57	B
T311	67	IIIc	E	3	yes	minimal	Dead	61	yes	22	B
T312	53	IIIb	E	3	yes	optimal	Dead	28	yes	28	B
T313	67	III	M	1	yes	optimal	Dead	22	yes	22	A
T314	76	IV	M	2	yes	optimal	Dead	15	yes	13	A
T315	70	IIIc	S	1	yes	optimal	Alive	136	no	136	A
T316	76	IV	S	1	yes	optimal	Alive	22	yes	22	A
T317	80	IIIc	S	2	yes	incomplete	Dead	6	yes	5	B
T318	61	IIIc	S	3	yes	unknown	Dead	5	yes	12	B

### DNA isolation and detection of genomic aberrations by array CGH

Tumor tissue was dissected from freshly cut 10 μm FFPE sections after confirming the tumor area by parallel haematoxylin and eosin staining. DNA was isolated as previously described [7, 12] using the QIAamp DNA micro kit (Qiagen, Hilden, Germany). DNA concentration and labelling quality were measured using NanoDrop 1000 Spectrophotometer (Thermo Scientific, Wilmington, DE, USA). Labelling with cyanine 3-dUTP (Cy3) and cyanine 5-dUTP (Cy5) nucleotides was performed using the array CGH labelling kit for oligo arrays according to the manufacturer’s protocol (Enzo Life Sciences, Farmingdale, NY, USA). DNA isolated from blood obtained from eighteen healthy females was pooled for use as a normal reference [13]. Free nucleotides were removed using the MinElute PCR Purification Kit (Qiagen). DNA was hybridized to 105 K whole genome Oxford design microarrays containing over 99.000 unique in situ synthesized 60-mer oligonucleotides (GPL8693, Agilent Technologies, Palo Alto, CA, USA), and hybridization was performed using the oligo array CGH/Chip-Chip Hybridization Kit 25 (Agilent). Each slide contained 2 arrays and one reference sample was hybridized per three tumor samples, as previously described [13]. Samples were hybridized overnight, were washed and subsequently scanned using a microarray scanner (Agilent, G2505B). Raw data of all the arrays performed are publicly available in the GEO database (accession number GSE24418).

### Data analysis

Array CGH quality was assessed by means of the median absolute deviation (MAD) of the log_2_ ratios of a chromosome arm without a breakpoint [14]. Whereas all MAD values of the copy number changes on the q-arm of chromosome two were between 0.17 and 0.43, the MAD value of one advanced stage sample was 0.70. This sample and its early stage match were therefore excluded from further analyses, leaving 16 early versus 16 advanced stage cases. With this set of 32 samples power analysis has been performed. Log2ratios were median normalized across array [13], wave patterns were smoothed [15]. Segmentation was performed by using circular binary segmentation (CBS), since this method has shown to substantially reduce the false positives caused by the local trends in the data [16, 17]. Mode normalization was performed on the segmented data prior to automated identification of losses, gains and amplifications [16]. Dimension reduction was achieved by summarizing into regions with a threshold of 0.01, in order to accept a maximum of 1 % information loss. The frequencies of aberrations in both groups were estimated, and the false discovery rate corrected p-values (Chi-square) of a difference in occurrence of aberrations between both groups were calculated after dividing the genome into regions [18, 19]. Using a FDR of 15 %, more than 50 % of discriminative aberrations will be detected [20]. To test whether advanced stage progresses from early stage, we tested whether the odds for aberrations in advanced stage samples were genome-wide higher than for the early stage samples (see supplementary materials for details). Weighted unsupervised clustering of called array CGH data was performed as described previously [21, 22]. Clusters found were correlated to overall and progression free survival using Kaplan-Meier survival analysis with log rank testing (SPSS software package version 17.0, Chicago, IL, USA). All other analyses were performed in the statistical framework R [23].

## Results

**Fig. 1:**
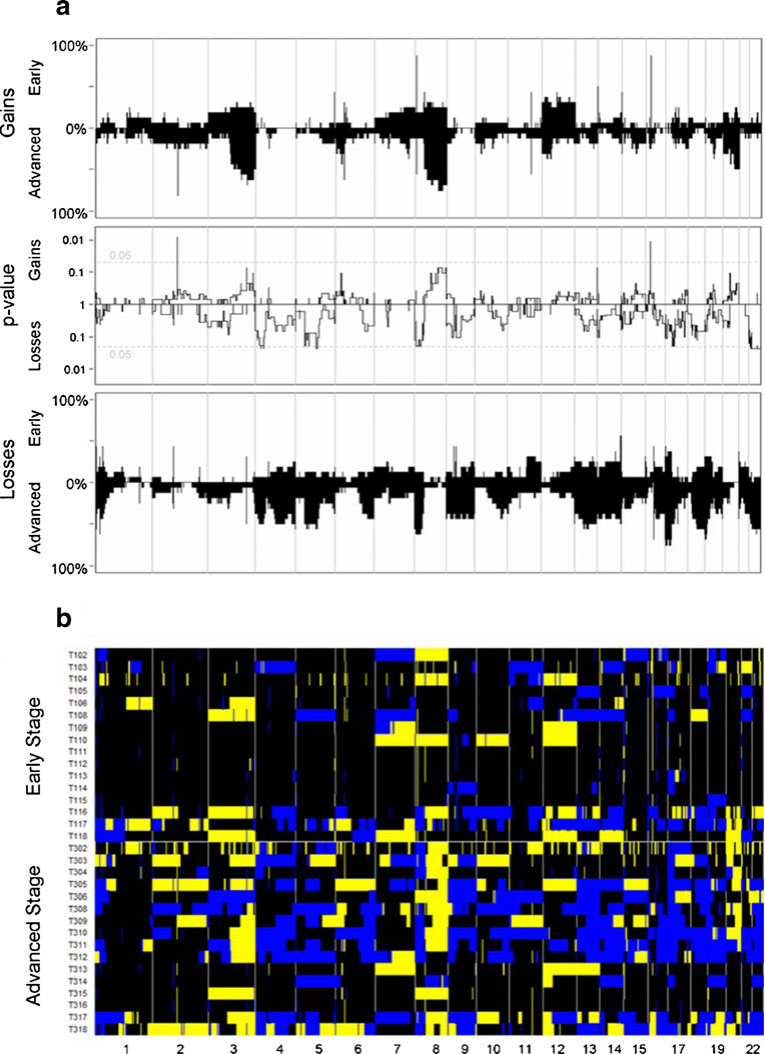
The frequencies of copy number gains in 16 early and 16 advanced stage ovarian cancer samples are plotted at the top of Panel A. The frequencies were tested for a difference between both stages and the false discovery rate corrected p-value is displayed. At the bottom of Panel A the analogue is shown for copy number losses. Panel B shows the array CGH profiles of all samples grouped per stage with *blue* indicating a loss, *black* a normal and *yellow* a gain in DNA copy number

**Fig. 2:**
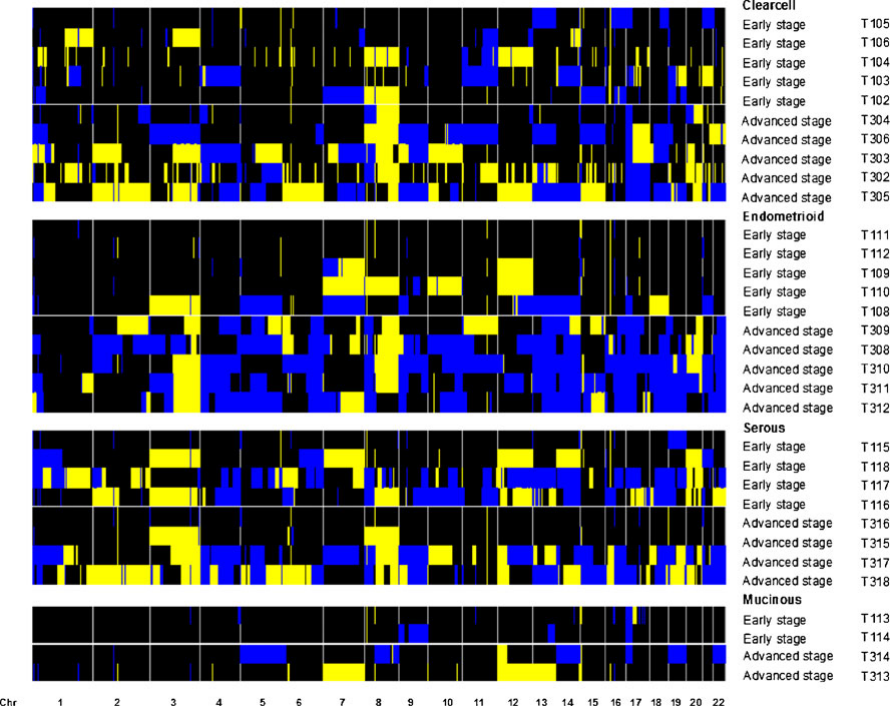
Array CGH profiles ordered per histological type and FIGO stage. *Blue* indicates a loss, *black* a normal and *yellow* a gain in DNA copy number

The patterns of aberrations in the advanced stage group mirrored those in the early stage group. Moreover, virtually all of the genomic aberrations were more frequent in the advanced stage group (Fig. 1a); the odds for aberrations were genome-wide significantly (*p* = 0.012 higher in advanced stage ovarian cancer than in early stage disease (See supplementary materials for details). When compared on patient level (Fig. 1b) the tumor profiles are heterogeneous. However, when stratified per FIGO stage according to histological subtype (Fig. 2) the profiles display more similarity. Furthermore, Fig. 2 shows that progression of aberrations is most pronounced in the clearcell and endometrioid subtypes.

When analysing the clinical and pathological characteristics of the tumors, we found the survival rate in patients with advanced stage ovarian cancer to be significantly lower than in patients with early stage cancer (*p* < 0.001), as expected. Contrastingly, stratification of the samples according to histological subtype yielded no significant difference in survival. However, patients with well-differentiated (grade one) tumors had significantly better survival than patients with intermediate and poorly (grades two and three) differentiated tumors combined (*p* = 0.044).

**Fig. 3:**
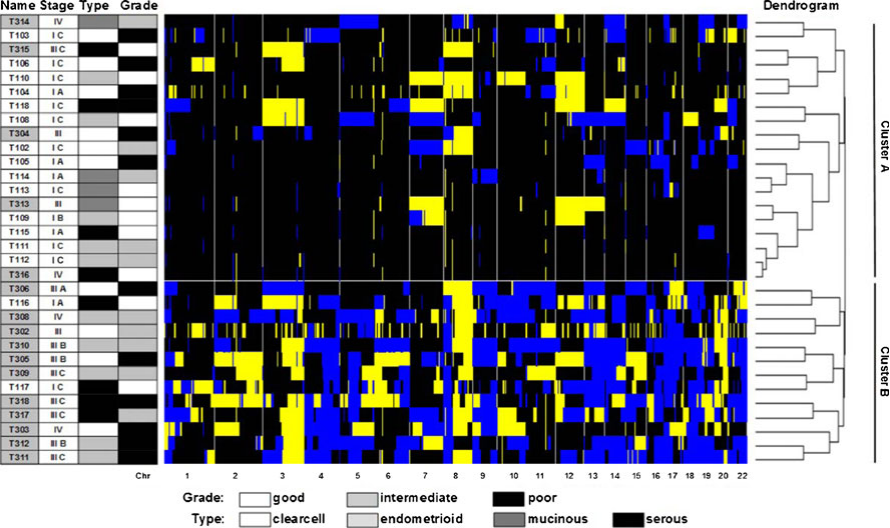
Heatmap of unsupervised clustering. *Blue* indicates a loss, *black* a normal, and *yellow* a gain in DNA copy number. The dendrogram on the right shows the similarity between the array CGH profiles. Left of the heatmap the tumor characteristics (FIGO stage, histological type and tumor differentiation grade) are displayed. A partition of the 32 ovarian cancer patients in cluster A and cluster B is found. In cluster A samples T104, T110, T304, T313, T314 and T316 had progression. In cluster B samples T117, T302, T303, T305, T308, T309, T310, T311, T312, T317 and T318 had progression

**Fig. 4:**
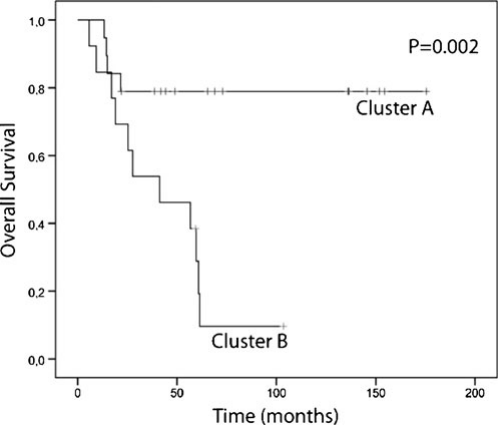
Kaplan-Meier plot based on cluster A (*n* = 19) and cluster B (*n* = 13) identified by unsupervised clustering of array CGH data. The p-value was calculated using the Mantel-Cox log rank test

In order to study if and how aberrations correlate to the clinical behaviour of the tumors, weighted clustering of called array CGH data was performed and yielded two distinct groups of 19 tumor samples in cluster A and 13 in cluster B (Fig. 3). Cluster A contained 5 advanced and 14 early stage tumors, of which four and two, respectively had a recurrence. Cluster B contained 11 advanced and two early stage tumors of which ten and one, respectively had a recurrence (Pearson Chi-square for distribution of FIGO stage between the clusters *p* = 0.001). With a mean survival of 142.0 months (95 %CI 112.7–171.2), patients in cluster A had a significantly (Mantel Cox log rank *p* = 0.002) better survival than patients in cluster B (43.0 months, 95 %CI 27.4–58.6) (Fig. 4).

## Discussion

Early and advanced stage ovarian carcinomas were compared with respect to chromosomal copy number aberrations measured by array CGH. Due to their distinct clinical presentation, we hypothesised that early and advanced stage ovarian cancer develop through separate molecular pathways. Aberrations were more frequent in advanced stage and there were virtually no aberrations in early stage that were not found in advanced stage. This finding suggests that advanced stage disease either progresses from early stage disease or from a common precursor lesion. This validates earlier findings in an independent data set using low resolution array CGH [6].

Unsupervised clustering of genomic aberrations significantly discriminated early from advanced stage samples. However, to differentiate molecular subtypes based on array CGH data, independent and high-resolution validation studies are necessary.

Depending on the scoring criteria, microsatellite instability (MSI) has been reported in 0–24 % of ovarian cancers [24–26], and little is known about their biological and clinical significance. However we did not test for MSI, so we can not exclude that in our samples there is MSI that could have influenced the outcome.

Ovarian carcinomas have been divided into subtypes by Shih and Kurman. [27] They propose a stratification based on clinical and histological characteristics in type I and type II tumors. Type I tumors are less frequent and are believed to be slow growing, generally confined to the ovary at diagnosis and genetically relatively stable. Histologically, type I tumors would consist of low-grade micropapillary serous carcinoma, mucinous, endometrioid, and clear cell carcinomas and would be associated with mutations in *KRAS*, *BRAF*, *PTEN*, and *beta-catenin*. Type II tumors would form the most common type of ovarian carcinomas and are rapidly growing, highly aggressive and genetically unstable. They would be high-grade serous carcinoma, malignant mixed mesodermal tumors and undifferentiated carcinomas and are associated with *TP53* mutations. As our study is based on FIGO stage 1 tumours we cannot compare cluster A and B with type I and II. However, we show that our study population (mainly consisting of type I tumours) can be further subdivided by array CGH into patients with good and poor overall survival.

The survival times differed significantly between both clusters. This finding suggests that the comparative genomic hybridization data of an ovarian carcinoma has the potency of being of clinical value in the future.

In conclusion, in this study we showed advanced stage ovarian cancer either progresses from early stage disease or from a common precursor lesion. We reject the hypothesis that the two stages might develop through distinct carcinogenic molecular events. Furthermore, when we divided patients in two groups solely on their genomic aberrations, we found these groups to differ significantly in survival. Since copy number analysis can be performed on easy to gain pre-treatment FFPE ovarian cancer tissue, array CGH data has the potency to identify patients with a worse prognosis.

## Electronic supplementary material

Online supporting information

Raw data of all the arrays performed are publicly available in the GEO database (accession number GSE24418).

S1 Details of the statistics to test whether odds for aberrations in advanced stage ovarian cancer are genome wide higher than in early stage. (JPEG 597 kb)

High resolution (TIFF 106 kb)

S2 Plots of the normalized and de-waved data with segmentation and calls of individual tumor samples. The blue lines represent the segments, the green bars the gains and the red bars the losses. The length of the bars represent the probability of the call. For the further analysis, calls were used with a probability of more than 50 %. The blue dots at the top of the figures indicate amplifications. These amplifications are handled as gains in the consecutive analysis. Balancing between the readability and accuracy of this Figure, we have used a lower resolution than in Figs. 1, 2 and 3. Therefore, some small aberrations seen in Figs. 1, 2 and 3 are not seen in this Figure. (PNG 4080 kb)
