# Supplementary figures and images for: Genomic aberrations relate early and advanced stage ovarian cancer
Source: Cell Oncol (Dordr). 2012 May 12;35(3):181–8. doi: 10.1007/s13402-012-0077-5 (PMC3396335; doi:10.1007/s13402-012-0077-5)

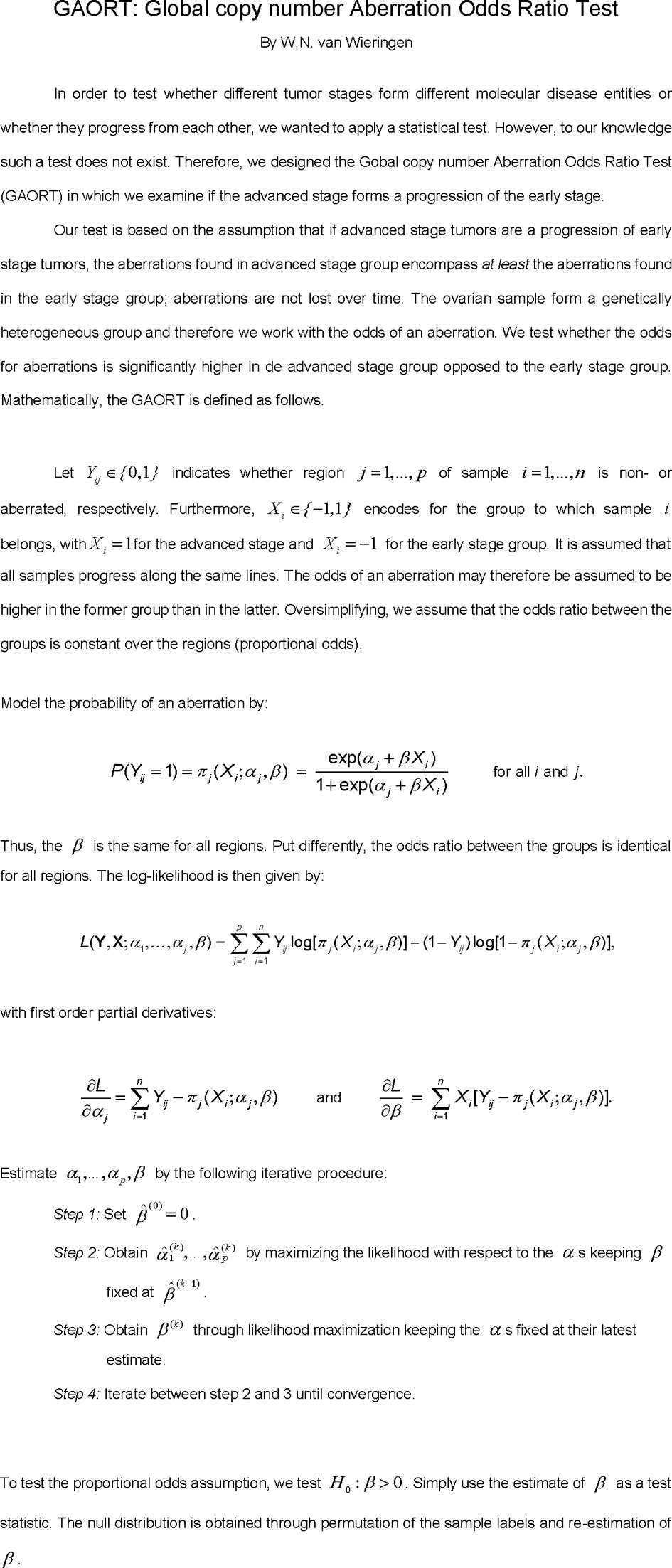

Supplement: Supplementary file 1 — Details of the statistics to test whether odds for aberrations in advanced stage ovarian cancer are genome wide higher than in early stage. (JPEG 597 kb) [file 13402_2012_77_Fig5_ESM.jpg]

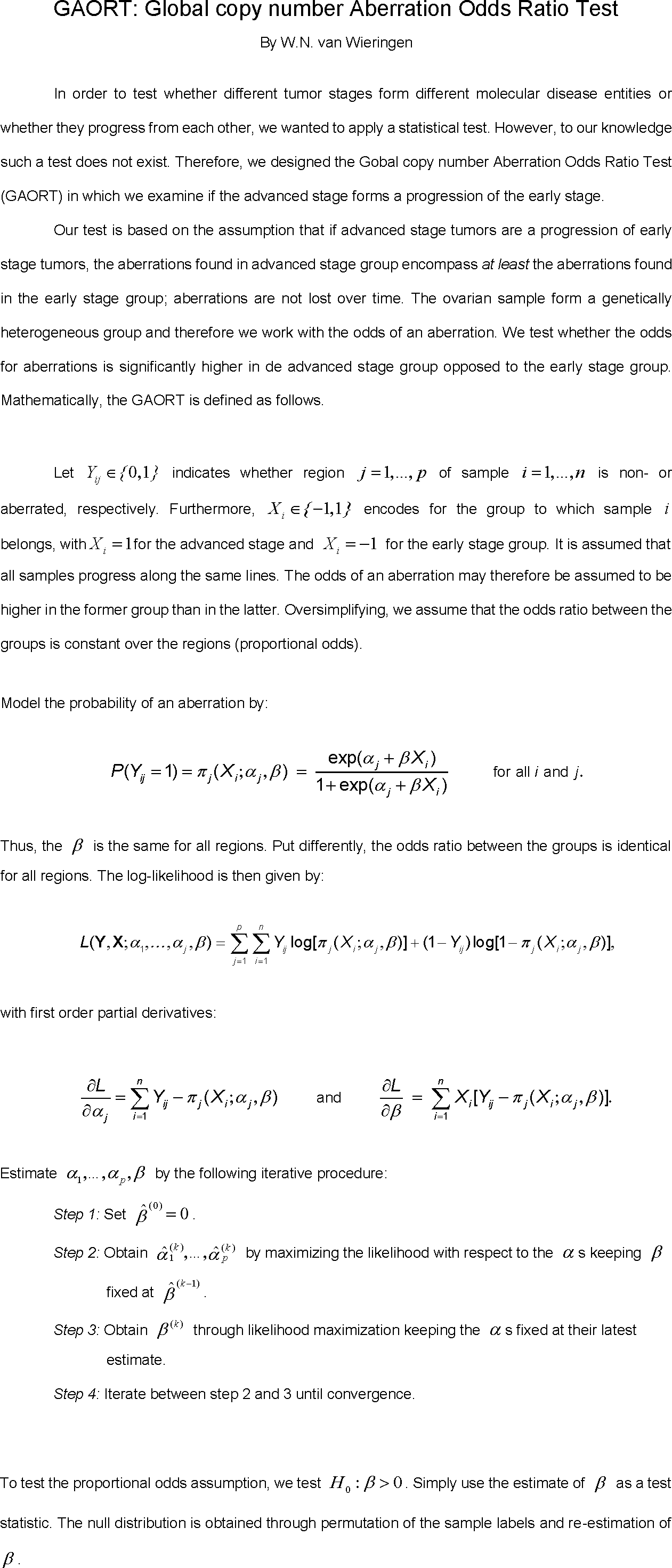

Supplement: Supplementary file 2 — High resolution (TIFF 106 kb) [file 13402_2012_77_MOESM1_ESM.tif]

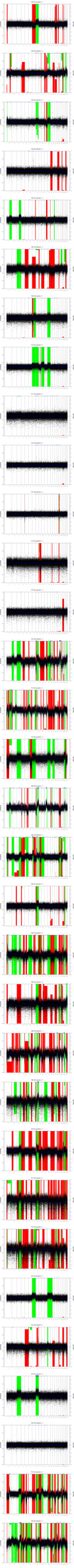

Supplement: Supplementary file 3 — Plots of the normalized and de-waved data with segmentation and calls of individual tumor samples. The blue lines represent the segments, the green bars the gains and the red bars the losses. The length of the bars represent the probability of the call. For the further analysis, calls were used with a probability of more than 50 %. The blue dots at the top of the figures indicate amplifications. These amplifications are handled as gains in the consecutive analysis. Balancing between the readability and accuracy of this Figure, we have used a lower resolution than in Figs. 1, 2 and 3. Therefore, some small aberrations seen in Figs. 1, 2 and 3 are not seen in this Figure. (PNG 4080 kb) [file 13402_2012_77_MOESM2_ESM.png]
